# Chloride of Sodium as a Substitute for Sulphate of Quinine, in Intermittent Fever

**Published:** 1852-01

**Authors:** 


*Chloride of Sodium as a Substitute for Sulphate of Quinine, in Intermittent Fever.*—Our readers doubtless remember that this substance was proposed some time ago, by Dr. Piorry, of Paris, as a remedy in intermittent fever, in evidence of the utility of which a large number of cases were adduced by him. He administers it in doses of two tablespoonsful once or twice daily, and asserts that it not only promptly arrests the paroxysms, but also exerts on the spleen as marked an influence as quinine does.

Professor Herrick, of Rush Medical College, has reported in the September number of the N. W. Medical and Surgical Journal, the results of several trials made with it, which go to corroborate the success obtained by M. Piorry. Prof. Herrick suggests that it acts by preventing the destruction of the blood globules (which takes place to a considerable extent in this disease,) and at the same time by furnishing the materials for the manufacture of a fresh supply of this constituent. Chloride of Sodium is known to possess the property of preserving the blood globules; it is an alterative and tonic, and is also claimed to possess a specific influence in arresting the exacerbations of intermittents.

He prescribes it in the dose of three to four drachms twice daily in mucilage. After the fever is checked, he gives it in smaller doses, say ten grains, with the same quantity of carb. ferri, twice or three times daily, as a tonic and corrective of the secretions of the alimentary tube.—*Ibid.*

Without any experience in regard to the febrifuge powers of the chloride of sodium, we can speak with great confidence of its efficacy in habitual constipation. Of all the laxatives we have ever tried, we have found this to act most pleasantly, uniformly, and naturally. Where the only object is to dislodge the contents of the lower bowels, it is all that physician or patient could desire.—Dyspeptics, sedentary persons, the subjects of hemorrhoids, all, in a word, who are troubled with costiveness, will find the remedy a mild and sure eccoprotic, emptying the bowels freely without nausea, irritation, or exhaustion. We direct it to be taken before breakfast, from two to three drachms, dissolved in two or three tumblers of cold water. The same dose continues to act from year to year, without diminution of effect.—[*Eds.*

